# Author Correction: Adjuvant nivolumab, capecitabine or the combination in patients with residual triple-negative breast cancer: the OXEL randomized phase II study

**DOI:** 10.1038/s41467-024-48359-1

**Published:** 2024-05-10

**Authors:** Filipa Lynce, Candace Mainor, Renee N. Donahue, Xue Geng, Greg Jones, Ilana Schlam, Hongkun Wang, Nicole J. Toney, Caroline Jochems, Jeffrey Schlom, Jay Zeck, Christopher Gallagher, Rita Nanda, Deena Graham, Erica M. Stringer-Reasor, Neelima Denduluri, Julie Collins, Ami Chitalia, Shruti Tiwari, Raquel Nunes, Rebecca Kaltman, Katia Khoury, Margaret Gatti-Mays, Paolo Tarantino, Sara M. Tolaney, Sandra M. Swain, Paula Pohlmann, Heather A. Parsons, Claudine Isaacs

**Affiliations:** 1 Division of Medical Oncology, Dana-Farber Cancer Institute, Boston, MA USA; 2 Breast Oncology Program, Dana-Farber Brigham Cancer Center, Boston, MA USA; 3 Harvard Medical School, Boston, MA USA; 4 MedStar Georgetown University Hospital, Washington, DC USA; 5 Center for Immuno-Oncology, Center for Cancer Research, National Cancer Institute, National Institutes of Health, Bethesda, MD USA; 6 Georgetown University, Washington, DC USA; 7 NeoGenomics, Durham, NC USA; 8 MedStar Washington Hospital Center, Washington, DC USA; 9 Tufts Medical Center, Boston, MA USA; 10 University of Chicago, Chicago, IL USA; 11 Hackensack University Medical Center, Hackensack, NJ USA; 12 University of Alabama at Birmingham, Birmingham, AL USA; 13 AstraZeneca, Arlington, VA USA; 14 Johns Hopkins Sidney Kimmel Cancer Center, Baltimore, MD USA; 15 Inova, Fairfax, VA USA; 16 The Ohio State University, Columbus, OH USA; 17 Present Address: AstraZeneca, Arlington, VA USA

**Keywords:** Breast cancer, Translational research, Cancer immunotherapy

Correction to: *Nature Communications* 10.1038/s41467-024-46961-x, published online 27 March 2024

The original version of the article file contained some inaccuracies in the main text. To avoid misinterpretation of the findings, the following corrections have now been made in the revised PDF and HTML versions of the article file.

**Abstract**.

The original sentence “*Here we show that a combination of nivolumab plus capecitabine leads to a greater increase in PIS from baseline to week 6 (91%) compared with nivolumab (47%) or capecitabine (53%) alone (log-rank p* *=* *0.08), meeting the pre-specified primary endpoint*.” has been replaced with “*Here we show that treatment with immunotherapy containing arms (nivolumab or a combination of nivolumab plus capecitabine) leads to an increase in PIS from baseline to week 6 compared with capecitabine alone, meeting the pre-specified primary endpoint*.”

**Results**.

In the section “Toxicity”, the original sentence “*There were 198 drug-related adverse events of any grade, with 29 (14.6%) reported in Arm A, 74 (37.4%) in Arm B, and 95 (48.0%) in Arm C (Table 2)”* has been replaced with “*There were 198 drug-related adverse events of any grade, with 29 (14.6%) reported in Arm A, 74 (37.4%) in Arm B, and 95 (48.0%) in Arm C. The most common drug-related adverse events in the safety analysis set are included in Table 2*.”

In the section “Prior Radiotherapy or BRCA1/2 Mutation Status and Landmark Immune Profile”, the sentences “*For this post-hoc analysis, all patients were combined due to the limited number of patients who had not received prior radiotherapy in each arm (4/11 in Arm A and Arm B, and 3/12 in Arm C)”* and “*For this post-hoc analysis, all patients were similarly combined due to the small number of patients with BRCA1/2 mutations enrolled (2/11 in Arm A, 1/11 in Arm B, and 0/12 in Arm C)*” have been rephrased respectively as “*For this post-hoc analysis, all patients were combined due to the limited number of patients who had not received prior radiotherapy in each arm (4 in Arm A and Arm B, and 3 in Arm C)”* and *“For this post-hoc analysis, all patients were similarly combined due to the small number of patients with BRCA1/2 mutations enrolled (2 in Arm A, 1 in Arm B, and 0 in Arm C)”*.

In the section “Detection of ctDNA”, there was a mistake in the final sentence “*It did not significantly differ by treatment arm, with 46% of ctDNA-evaluable patients in Arm A found to be ctDNA-positive at landmark compared to 33.3% and 30% in Arms B and C, respectively (Supplementary Table 10).”* that has been corrected as *“It did not significantly differ by treatment arm, with 46% of ctDNA-evaluable patients in Arm A found to be ctDNA-positive at landmark compared to 33.3% and 23% in Arms B and C, respectively (Supplementary Table 10).”*.

**Discussion**.

The sentence “*The study met the pre-specified primary endpoint, with patients treated with immunotherapy containing regimens (arms A and C) experiencing a greater increase at week 6 versus baseline in a peripheral immunoscore (immunoscore #1) compared to patients treated with chemotherapy alone (Arm B)*” has been rephrased as “*The study met the pre-specified primary endpoint, with patients treated with immunotherapy containing regimens (arms A and C) experiencing an increase at week 6 versus baseline in a peripheral immunoscore (immunoscore #1) compared to patients treated with chemotherapy alone (Arm B)*.”

