# Supplementary material for: Adjuvant nivolumab, capecitabine or the combination in patients with residual triple-negative breast cancer: the OXEL randomized phase II study
Source: Nat Commun. 2024 Mar 27;15:2691. doi: 10.1038/s41467-024-46961-x (PMC10973408; doi:10.1038/s41467-024-46961-x)
Supplement: Supplementary file 3 — Reporting Summary [file 41467_2024_46961_MOESM3_ESM.pdf]

nature portfolio

Corresponding author(s): Fi lipa Ly n c e

Last updated by author(s): March 14, 2024

Reporting Summary

Nature Portfolio wishes to improve the reproducibility of the work that we publish. This form provides structure for consistency and transparency in reporting. For further information on Nature Portfolio policies, see our Editorial Policies and the Editorial Policy Checklist.

Statistics

For all statistical analyses, confirm that the following items are present in the figure legend, table legend, main text, or Methods section.

n/a	Confirmed	
[ ]	[x]	The exact sample size (*n*) for each experimental group/condition, given as a discrete number and unit of measurement
[ ]	[x]	A statement on whether measurements were taken from distinct samples or whether the same sample was measured repeatedly
[ ]	[x]	The statistical test(s) used AND whether they are one- or two-sided<br>*Only common tests should be described solely by name; describe more complex techniques in the Methods section.*
[ ]	[x]	A description of all covariates tested
[ ]	[x]	A description of any assumptions or corrections, such as tests of normality and adjustment for multiple comparisons
[ ]	[x]	A full description of the statistical parameters including central tendency (e.g. means) or other basic estimates (e.g. regression coefficient) AND variation (e.g. standard deviation) or associated estimates of uncertainty (e.g. confidence intervals)
[ ]	[x]	For null hypothesis testing, the test statistic (e.g. *F*, *t*, *r*) with confidence intervals, effect sizes, degrees of freedom and *P* value noted<br>*Give P values as exact values whenever suitable.*
[x]	[ ]	For Bayesian analysis, information on the choice of priors and Markov chain Monte Carlo settings
[x]	[ ]	For hierarchical and complex designs, identification of the appropriate level for tests and full reporting of outcomes
[x]	[ ]	Estimates of effect sizes (e.g. Cohen's *d*, Pearson's *r*), indicating how they were calculated

Our web collection on statistics for biologists contains articles on many of the points above.

Software and code

Policy information about availability of computer code

Data collection	Data collection was performed with electronic case report forms built specifically for this trial. There was no specific software used.
Data analysis	Statistical analyses were performed using SAS Software Version 9.4 (SAS Inc, Cary, NC), RStudio (Version 1.4.1106) and Graph Pad Prism (Version 9, Graph Pad Software, La Jolla, CA).

For manuscripts utilizing custom algorithms or software that are central to the research but not yet described in published literature, software must be made available to editors and reviewers. We strongly encourage code deposition in a community repository (e.g. GitHub). See the Nature Portfolio guidelines for submitting code & software for further information.

Data

Policy information about availability of data

All manuscripts must include a data availability statement. This statement should provide the following information, where applicable:

- Accession codes, unique identifiers, or web links for publicly available datasets
- A description of any restrictions on data availability
- For clinical datasets or third party data, please ensure that the statement adheres to our policy

Summarized clinical data , peripheral immune subset data and the original clinical trial protocol are provided as Supplementary Information. Additional de-identified participant data are available for academic purposes on request from the corresponding author, Dr. Filipa Lynce (filipa_lynce@dfci.harvard.edu). According to Georgetown IRB authorization based on patients' consent to share genomic data, 29 patients out of 38 with genomic data available consented to have their data deposited. The WES data of 29/38 patients have been deposited at the European Genome-phenome Archive (EGA), which is hosted by the EBI and the CRG, under accession number EGAS50000000222 . Further information about EGA can be found at https://ega-archive.org and "The European Genome-phenome Archive of human data consented for biomedical research". Controlled access is required to ensure that data use is not for profit or commercial purposes. Data are available by submitting a data access request via the EGA portal (see https://ega-archive.org/access/request-data/how-to-request-data/ for detailed guidance). Source data are provided in this paper.

Research involving human participants, their data, or biological material

Policy information about studies with human participants or human data. See also policy information about sex, gender (identity/presentation), and sexual orientation and race, ethnicity and racism.

Reporting on sex and gender	This study was open for all patients irrespective of sex and gender. All 45 patients enrolled were women.
Reporting on race, ethnicity, or other socially relevant groupings	This study was open for all patients irrespective of race, ethnicity or social group.
Population characteristics	All patients had a confirmed diagnosis of triple negative breast cancer. Any patient age 18 or older was eligible to participate. The mean age was 51 years old.
Recruitment	Patients were enrolled at MedStar Georgetown University Hospital, MedStar Washington Hospital Center, University of Chicago, University of Alabama Birmingham, and Hackensack University Medical Center. All patients provided informed consent. Patients were referred to large academic centers to enroll in this trial which could have led to a selection bias of patients who could go to these large centers.
Ethics oversight	This study was approved by the MedStar Georgetown University Hospital Institutional Review Board

Note that full information on the approval of the study protocol must also be provided in the manuscript.

Field-specific reporting

Please select the one below that is the best fit for your research. If you are not sure, read the appropriate sections before making your selection.

☒ Life sciences ☐ Behavioural & social sciences ☐ Ecological, evolutionary & environmental sciences

For a reference copy of the document with all sections, see nature.com/documents/nr-reporting-summary-flat.pdf

Life sciences study design

All studies must disclose on these points even when the disclosure is negative.

Sample size	The sample size of 45 patients with 15 patients per arm had an 85% power to detect an effect size of 1 (the difference of the change in peripheral immunoscore from landmark to week 6 between two arms divided by the standard deviation) at 5% significance level.
Data exclusions	No data were excluded from the analysis
Replication	N/A as these were results from a single clinical trial.
Randomization	Random allocation
Blinding	Investigators were blinded to the allocation process but given the nature of the drug received, they knew what treatment patients received. That doesn't impact results of ctDNA, peripheral immunoscore or recurrence.

Reporting for specific materials, systems and methods

We require information from authors about some types of materials, experimental systems and methods used in many studies. Here, indicate whether each material, system or method listed is relevant to your study. If you are not sure if a list item applies to your research, read the appropriate section before selecting a response.

Materials & experimental systems

n/a	Involved in the study
☒	☐ Antibodies
☒	☐ Eukaryotic cell lines
☒	☐ Palaeontology and archaeology
☒	☐ Animals and other organisms
☐	☒ Clinical data
☒	☐ Dual use research of concern
☒	☐ Plants

Methods

n/a	Involved in the study
☒	☐ ChIP-seq
☐	☒ Flow cytometry
☒	☐ MRI-based neuroimaging

Clinical data

Policy information about clinical studies

All manuscripts should comply with the ICMJEguidelines for publication of clinical research and a completedCONSORT checklist must be included with all submissions.

Clinical trial registration: NCT03487666

Study protocol: Full trial protocol submitted with manuscript

Data collection: Patients were recruited between August 2018 and June 2021. Data were collected during the same period. Survival and recurrence data collection continued after completion of study enrollment. Data were collected by research team at each individual institution and analyzed by biostatisticians at Georgetown who received unidentified data.

Outcomes: The primary endpoint was to assess the effects of nivolumab, capecitabine, or the combination on the peripheral immune profile. The peripheral immune profile was defined from cryopreserved PBMCs collected from patients at landmark, 6 weeks, and 12 weeks examined by multicolor flow cytometry using 30 markers in 4 panels to identify 158 peripheral immune cell subsets with known biologic function (Supplementary Table 3).

Detection of ctDNA at landmark, at 6 weeks, and at 12 weeks was a secondary endpoint. Archival tumor tissue was obtained preferentially from definitive breast cancer surgery, or from initial diagnostic biopsy if tissue was insufficient. Formalin-fixed paraffin-embedded (FFPE) tissue block or 10-20 unstained slides and hematoxylin and eosin stain (H&E) slide from each patient were sent to Invitata, Inc. (Durham, NC), where DNA was extracted and WES was performed as previously described. The unique somatic mutation profile of each tumor was used to design a personalized RaDaR TM assay to detect ctDNA in plasma samples from each patient. Additional secondary endpoints included incidence of toxicity using the NCI CTCAE v.4.0, OS and iDFS, and association between ctDNA and peripheral immune profile with recurrence and survival. iDFS was defined as the time from date of randomization to the date of first invasive disease recurrence, second invasive primary cancer (breast or not), or death from any cause. OS was defined as the time from date of randomization to death from any cause.

Flow Cytometry

Plots

Confirm that:

☒ The axis labels state the marker and fluorochrome used (e.g. CD4-FITC).

☒ The axis scales are clearly visible. Include numbers along axes only for bottom left plot of group (a 'group' is an analysis of identical markers).

☒ All plots are contour plots with outliers or pseudocolor plots.

☒ A numerical value for number of cells or percentage (with statistics) is provided.

Methodology

Sample preparation: Cryopreserved PBMCs collected from patients at landmark, 6 and 12 weeks

Instrument: Flow cytometry files were acquired on an LSRFortessa (BD Biosciences, Franklin Lakes, NJ) equipped with 5 lasers.

Software: Flow cytometry files were analyzed using FlowJo v.9.9.6 (FlowJo LLC, Ashland, OR) for Macintosh, with nonviable cells excluded and negative gates based on fluorescence-minus-one controls

Cell population abundance: The frequency of all subsets was calculated as a percentage of PBMCs to eliminate any bias that might occur in the smaller populations with fluctuations in leukocyte subpopulations.

Gating strategy: *Describe the gating strategy used for all relevant experiments, specifying the preliminary FSC/SSC gates of the starting cell population, indicating where boundaries between "positive" and "negative" staining cell populations are defined.*

☒ Tick this box to confirm that a figure exemplifying the gating strategy is provided in the Supplementary Information.
